# Case Report: AMSAN variant of GBS complicating immediate postpartum period with severe dysautonomia leading to Posterior Reversible Encephalopathy Syndrome

**DOI:** 10.12688/f1000research.147190.2

**Published:** 2024-07-01

**Authors:** Koushik Ramachandra, Hitha Almengada, Meghana Deepak Madi

**Affiliations:** 1 Department of General Medicine, Kasturba Medical College, Mangalore, Manipal Academy of Higher Education, Manipal, India

**Keywords:** Guillain Barre Syndrome (GBS), Acute Motor- Sensory Axonal Neuropathy (AMSAN), Posterior Reversible Encephalopathy Syndrome (PRES), Plasma Exchange, Post Partum, Intrauterine Death

## Abstract

A 20’s primiparous woman, following spontaneous expulsion of intrauterine death of the fetus at 30 weeks of gestation, presented on post-partum day 8 with acute onset flaccid quadriparesis and breathing difficulty, which had rapidly progressed to involve the legs on day 3 up to her upper limbs on post-partum day 5. Following examination, Guillain Barre Syndrome (GBS) with ascending diaphragmatic involvement was diagnosed, and plasma exchange was initiated. She developed raised blood pressure, headache, sudden onset visual loss with 2 episodes of generalized seizures on post-partum day 14. Brain MRI and clinical suspicion helped diagnose Posterior Reversible Encephalopathy Syndrome (PRES). The patient was treated with anticonvulsants and antihypertensive agents. She regained her vision over the next two days, completed the treatment for GBS, and made a good recovery with independence for advanced activities of daily living on follow-up.

## Introduction

The risk of developing Guillian Barre Syndrome (GBS) increases after delivery, particularly during the first two weeks of puerperium. ^1^

Here, we present a case of Acute Motor- Sensory Axonal Neuropathy (AMSAN), the rare and most severe form of GBS, complicating the postpartum period in our patient. Acute Motor Sensory Axonal Neuropathy (AMSAN)-type GBS is characterized by sensory and motor fiber axonal degeneration. ^2^

Prevention of severe axonal damage in the early stages of the disease promotes a favourable long-term outcome. ^3^ Preganglionic sympathetic axonal demyelination or postganglionic axonal degeneration causes blood pressure fluctuations in GBS patients. ^4^

Our patient with AMSAN-type GBS developed severe dysautonomia and accelerated hypertension, which ultimately led to Posterior Reversible Encephalopathy Syndrome (PRES). Timely care aided in her favourable outcomes.

## Case report

A 20’s female with no prior medical or neurological disorders, sustained an antepartum stillbirth, following spontaneous expulsion. She presented to the emergency department on postpartum day 8 with chief complaints of inability to move both lower limbs for 5 days, inability to raise her arms for 3 days, and difficulty in breathing since that morning. Her symptoms started immediately postpartum and had rapidly progressed over the last five days. On examination, she was conscious, well-oriented, and afebrile, with stable vitals. She was tachypneic, dyspneic with oxygen saturation of 98% on ambient air and had a single breath count of 13. Neurological examination demonstrated flaccidity in all upper and lower limb muscles with a power of 2/5 for the proximal muscles, 4/5 for the distal muscles of the upper limb, and 1/5 power for both proximal and distal muscles of the lower limb, bilaterally (as per the Medical Research Council scale grading for muscle strength). The patient also demonstrated areflexia with mute plantar reflex. There were features suggestive of bilateral LMN facial nerve and bulbar palsy. The patient was noted to have symmetrical glove and stocking-type loss of pin prick sensation in the limbs, loss of temperature sensation with normal proprioception and vibration senses. No features of autonomic disturbances were observed. At the end of the examination, GBS with ascending diaphragmatic involvement was suspected and confirmed by Nerve Conduction Velocity (NCV) studies. NCV, as shown in Figure 2 and Figure 3, revealed the AMSAN variant of GBS, with reduced amplitudes of CMAPs and SNAPs, and absence of non-anatomical conduction blocks. CSF analysis did not reveal albumin-cytologic dissociation/cellular reaction. Complete blood counts did not show leucocytosis. Serum electrolytes were normal. Anti-nuclear antibodies, anti-neutrophil cytoplasmic antibodies by immunofluorescence were negative. She tested negative for HIV, Hepatitis B and Hepatitis C infection.

**Figure 1.**
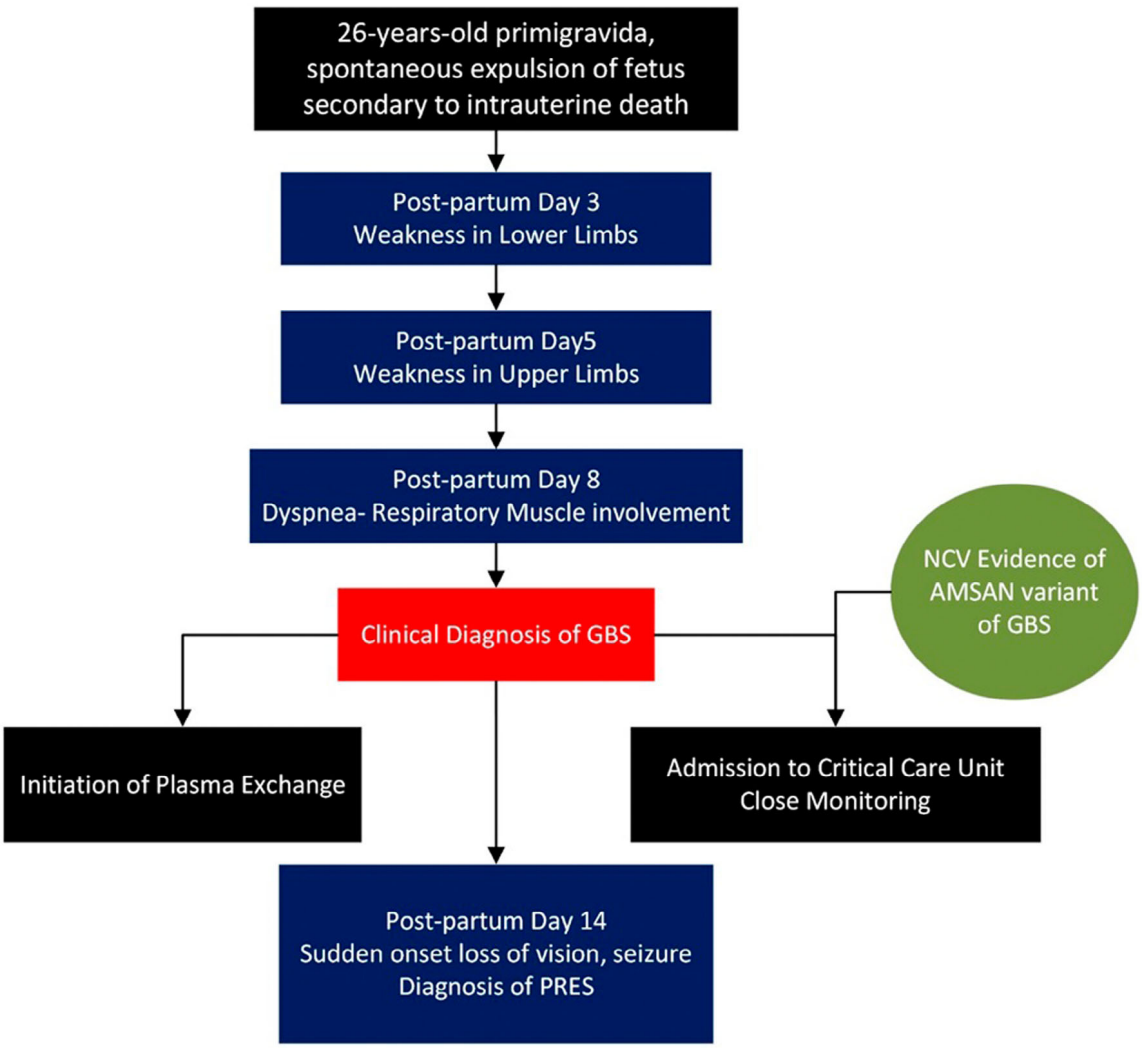
Timeline of events.

**Figure 2.**
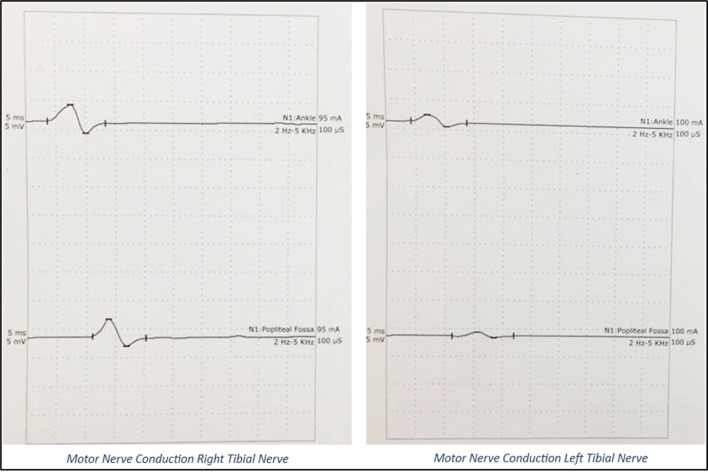
CMAPs on Nerve Conduction Study: Grossly reduced amplitudes of CMAPs.

**Figure 3.**
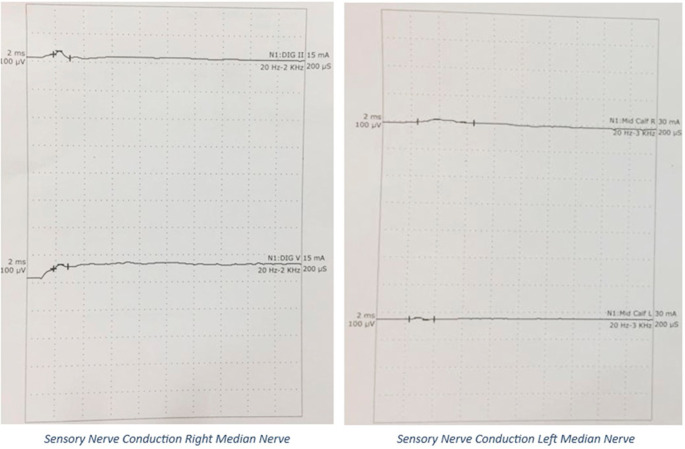
SNAPs on Nerve Conduction Study: Absent SNAPs.

**Figure 4.**
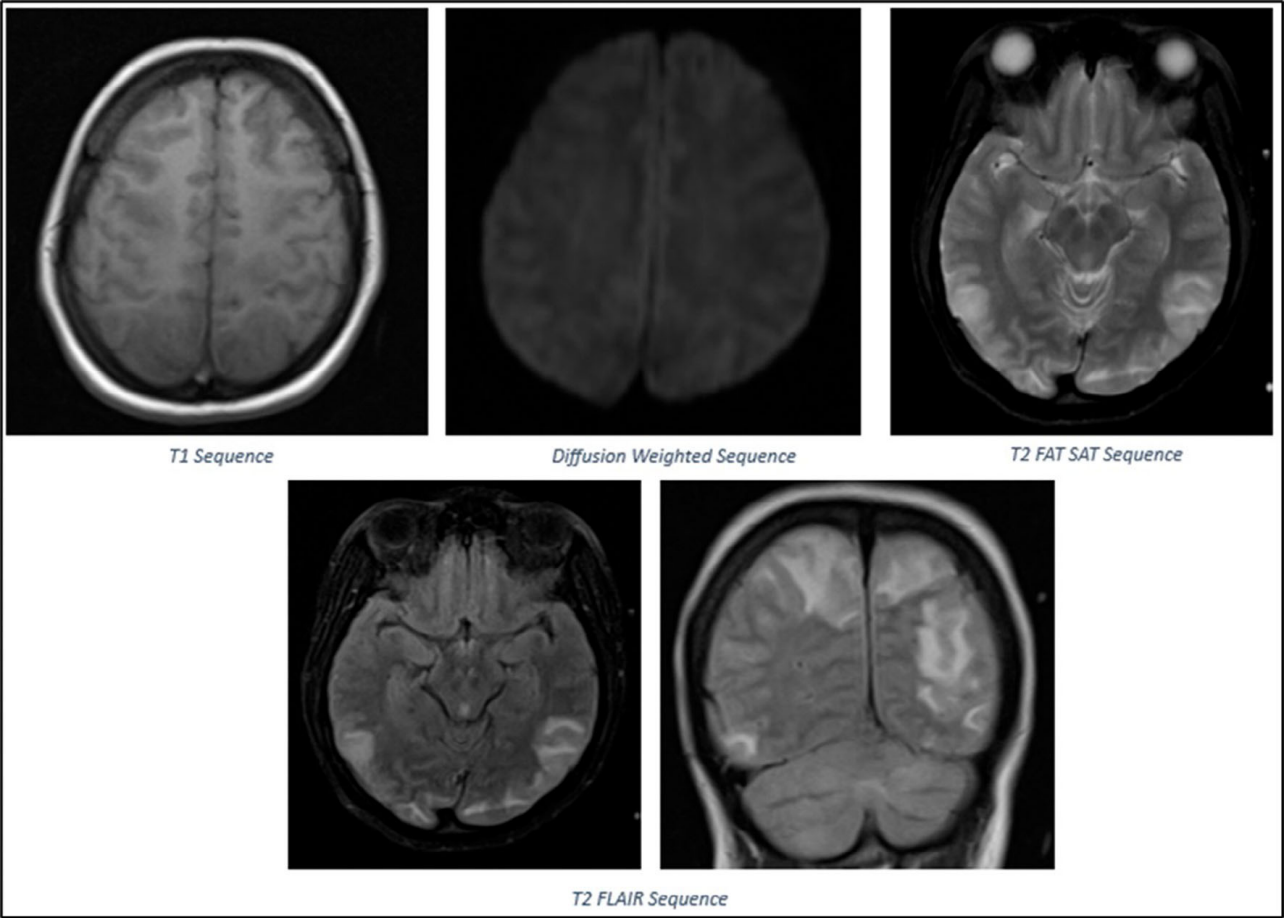
MRI Brain Findings: Cortical and Subcortical T2/FLAIR hyperintensities with oedema in bilateral high frontal, posterior parietal, occipital regions, and bilateral cerebellar hemispheres consistent with PRES.

The patient was transferred to intensive care unit and started on emergency therapeutic plasmapheresis. The patient was intubated, and lung-protective ventilation was initiated in view of worsening respiratory distress. On postpartum day 14, she developed sudden onset loss of vision, headache, raised blood pressure of 200/110 mmHg and two episodes of generalized tonic-clonic seizures. Ophthalmic examination revealed papilledema, and absent perception of light bilaterally. Brain MRI was noted to have cortical and subcortical T2/FLAIR hyperintensities with edema in the bilateral high frontal, posterior parietal, occipital regions, and bilateral cerebellar hemispheres, consistent with posterior reversible encephalopathy syndrome (PRES), as shown in Figure 4. She was treated with antiseizure medications and antihypertensives. The patient regained vision over the next two days. The patient completed 5 cycles of plasmapheresis. Simultaneously, the patient underwent limb and chest physiotherapy. The patient was discharged after one month of hospitalization when she was able to walk with support. The timeline of the patient’s course in the hospital is presented in Figure 1.

## Discussion

GBS is a set of autoimmune diseases characterized by polyradiculoneuropathy. It is typically preceded by infectious diseases or immunological stimulation that triggers autoimmune reactions in peripheral nerves. ^5^ While demyelinating variant is the most common subtype observed worldwide, axonal variant remains the most common variant in India. ^2^^,^^6^^,^^7^ Our patient did not report infection days or weeks prior to symptom onset. Her symptoms developed postpartum, and there was no history or records during the antenatal period that were suggestive of GBS prior to delivery. Moreover, there is no evidence to suggest that our patient had GBS prior to delivery, which may have resulted in antepartum stillbirth. A thorough history ruled out the possibility of GBS in the last trimester of her pregnancy, and that her current condition could either be a relapse or a continuation of the disease process post-delivery.

GBS can develop in any trimester and postpartum period, but especially in the third trimester and first two weeks after delivery, ^9^ as seen in our patient who developed symptoms 3 days after delivery. A three-year retrospective observational study of medical records of all pregnant and postpartum women diagnosed with GBS based on the clinical, laboratory, and electrodiagnostic criteria showed that 50% of the women presented during the postpartum period. ^10^ Several pathophysiological factors contribute to the development of the various GBS subtypes. In AMSAN, the target peptides are located on sensory and motor neuronal axons. ^8^ Throughout the postpartum period, there is an increase in pro-inflammatory cytokines, which may account for the increased incidence of the disease. GBS is frequently reported to worsen during the postpartum period because of an increase in delayed-type hypersensitivity, which could also be the reason for our patient’s rapid deterioration of symptoms, in addition to having the AMSAN variant. ^9^

Variants of GBS, as well as the ensuing involvement of respiratory muscles and autonomic dysfunction, contribute to maternal mortality. ^10^ Approximately 20% of GBS patients experience respiratory failure that requires mechanical ventilation. ^11^^,^^12^ By the time our patient reached our centre, she had already progressed to respiratory muscle involvement. It is recommended to routinely measure respiratory function, since all individuals with respiratory insufficiency do not have dyspnea. A single breath count of ≤19 indicates the necessity for mechanical ventilation, and other respiratory parameters may include the use of accessory respiratory muscles, vital capacity, and maximal inspiratory and expiratory pressure. ^11^ At the time of presentation, our patient had a single breath count of 13 with accessory respiratory muscles requiring respiratory support. Early intravenous immunoglobulin (IVIG) or plasma exchange therapy has been shown to be effective and is crucial in patients with rapidly worsening weakness, ^3^ and needs to be initiated before irreparable nerve damage develops. As observed in our patient during the first week, cerebrospinal fluid analysis did not reveal the expected albumin-cytologic dissociation. ^5^

Uncertainty surrounds the mechanism of PRES, a neurological disorder that typically manifests as visual impairment, seizures, and encephalopathy, in the setting of GBS. In neuroimaging, it is characterized by bilateral parietal and occipital cortical/subcortical vasogenic edema, followed by frequent involvement of additional regions. ^14^ Vasogenic edema may be caused by hypertension due to autonomic dysfunction, which exceeds the limits of cerebrovascular autoregulation. ^15^ Dysautonomia is observed in approximately 70% of patients with GBS. Altered sympathetic activity resulting in severe hypertension, high levels of cytokines in serum and CSF which alter capillary permeability may contribute to development of PRES. ^13^^,^^17^ Blood pressure variability is a key trait of GBS. The observed variances may be explained by changes in the feedback control caused by preganglionic sympathetic axonal demyelination or postganglionic axonal degeneration. ^4^ In-hospital mortality was greater, and functional outcomes at hospital discharge were poorer in patients with autonomic instability. ^18^ PRES has been reported even in patients with normal blood pressures. ^19^ This shines light on alternative pathophysiologic mechanisms surrounding GBS and PRES. Dyselectrolytemia, especially hyponatremia has been associated with PRES in patients who are normotensive. ^18^ Treatment with IVIG with possible mechanisms of vasogenic edema, cerebrovascular endothelial dysfunction, cerebral vasospasm, serum hyperviscosity, intravascular hypercoagulopathy, and platelet hyperactivity, has been reported as a known trigger for PRES. ^20^ Also, imaging of the brain is not routinely preformed in patients with GBS as it is believed to be a peripheral nerve disorder. Presence of systemic symptoms like fever, weight loss, history of immunocompromised state, history of malignancy, altered consciousness, focal deficits, sudden-onset headache, new headache in patients over the age of 50 years, papilloedema warrant neuroimaging. ^19^

Our patient with the AMSAN variant developed severe dysautonomia on post-partum day 14 with accelerated hypertension leading to PRES. Paneyala S, et al. described a similar case of a woman who developed GBS shortly after delivery, but later experienced seizures due to PRES. ^16^

The fundamentals of GBS care in pregnancy and the postpartum period include timely detection, multidisciplinary input, and swift plasmapheresis or IVIG administration. Dysautonomia frequently complicates the course of GBS. Early recognition improves outcomes for both the mother and the fetus. ^1^

## Consent to participate

Written informed consent was given by the patient.

## Consent to publish

Written consent from the patient was taken for publishing the case after de-identifying and anonymizing all the patient identifiers.

## Data Availability

“No data are associated with this article.”
