# Supplementary material for: Case Report: AMSAN variant of GBS complicating immediate postpartum period with severe dysautonomia leading to Posterior Reversible Encephalopathy Syndrome
Source: F1000Res. 2024 Jul 1;13:327. Originally published 2024 Apr 23. [Version 2] doi: 10.12688/f1000research.147190.2 (PMC11384208; doi:10.12688/f1000research.147190.2)
Supplement: Peer review history

# Reviewer report: Reviewer response for version 2

**DOI:** 10.5256/f1000research.168369.r321853

**Published:** 2024-09-09

**Authors:** Dr. Radha M G

**Affiliations:** 1 Ramaiah Memorial Hospital, Bengaluru, India

Rare case and authors have sufficiently explained and justified the diagnosis which is challenging. Timely management has resulted in good recovery and outcome .

 Only suggestion would be the time line need to be specified for neurological recovery and chronology of recovery.

 Both GBS and PRESS are neurological emergencies, timely evaluation and management resulting in good outcome is appreciable .

Are enough details provided of any physical examination and diagnostic tests, treatment given and outcomes?

Yes

Is the case presented with sufficient detail to be useful for other practitioners?

Yes

Is sufficient discussion included of the importance of the findings and their relevance to future understanding of disease processes, diagnosis or treatment?

Yes

Is the background of the case’s history and progression described in sufficient detail?

Yes

Reviewer Expertise:

Critical care medicine

I confirm that I have read this submission and believe that I have an appropriate level of expertise to confirm that it is of an acceptable scientific standard.

# Reviewer report: Reviewer response for version 2

**DOI:** 10.5256/f1000research.168369.r310567

**Published:** 2024-08-24

**Authors:** Josef Finsterer

**Affiliations:** 1 Neurology Department, Neurology & Neurophysiology Center, Vienna, Austria

AMSAN variant of GBS complicating immediate postpartum period with severe dysautonomia leading to Posterior Reversible Encephalopathy Syndrome.

 Before attributing arterial hypertension to dysautonomia in Guillain barre syndrome, all other causes of art. hypertension must be thoroughly ruled out.

 There is need that autonomic comprehensive testing is performed to document that the autonomic nervous system is indeed involved in the disease.

 There is also a need to repeat nerve conductions studies to document either progression of the disease or spontaneous recover y or recovery from treatment.

Are enough details provided of any physical examination and diagnostic tests, treatment given and outcomes?

Yes

Is the case presented with sufficient detail to be useful for other practitioners?

Yes

Is sufficient discussion included of the importance of the findings and their relevance to future understanding of disease processes, diagnosis or treatment?

No

Is the background of the case’s history and progression described in sufficient detail?

Yes

Reviewer Expertise:

NMD

I confirm that I have read this submission and believe that I have an appropriate level of expertise to state that I do not consider it to be of an acceptable scientific standard, for reasons outlined above.

# Response

**Published:** 2024-08-24

**Authors:** Koushik Ramachandra

**Affiliations:** General Medicine, Kasturba Medical College, Mangalore, Manipal Academy of Higher Education, Manipal, Mangalore, Karnataka, India

Sir,

 We thank you for reviewing our case report.

 We would like to put forth our view for the concerns raised.

1. Need to rule out arterial hypertension prior to attributing dysautonomia as etiology: Sir our patient was not noted to have elevated blood pressures during her antenatal visits, admission at the other healthcare center wherein her symptoms started and also on admission at our center. The sudden change in pressure was not associated with renal dysfunction either. It was thereby attributed to the ongoing neurological disease. 
2. Need for detailed autonomic function assessment: We agree with the need for a detailed assessment. Such an assessment could not be performed given the critical nature of her course, ongoing organ supports (ventilator requirement), rapid changes in fluid volumes (ongoing plasma exchange cycles).
3. Need for repeat NCS: We agree with the need for repeat studies. She improved during the course of her hospital stay gradually (no abrupt improvement) over 1 month, which would bring the total course of illness to ~6 weeks. We thereby deduced that it was attributable to the treatment and also the expected course of the disease and did not repeat the NCS as she had recovered clinically.

Kindly do consider our view.

# Reviewer report: Reviewer response for version 2

**DOI:** 10.5256/f1000research.168369.r297791

**Published:** 2024-07-03

**Authors:** Ivana Berisavac

**Affiliations:** 1 University of Belgrade, Belgrade, Serbia

The authors answer the questions. I approved the article.

Are enough details provided of any physical examination and diagnostic tests, treatment given and outcomes?

No

Is the case presented with sufficient detail to be useful for other practitioners?

Yes

Is sufficient discussion included of the importance of the findings and their relevance to future understanding of disease processes, diagnosis or treatment?

Partly

Is the background of the case’s history and progression described in sufficient detail?

Partly

Reviewer Expertise:

GBS, Status epilepticus, myasthenia gravis crisis

I confirm that I have read this submission and believe that I have an appropriate level of expertise to confirm that it is of an acceptable scientific standard.

# Reviewer report: Reviewer response for version 1

**DOI:** 10.5256/f1000research.161356.r288637

**Published:** 2024-06-24

**Authors:** Ivana Berisavac

**Affiliations:** 1 University of Belgrade, Belgrade, Serbia

There is no previous medical history of patient. We don't know did she have other diseases? The author only says that she was not febrile. It means nothing.

 What are her laboratory tests?

 On what basis was the infection ruled out?

 What is the result of lumbar puncture?

 Was cerebrospinal neuro panel performed?

 Where Tu markers, immunological analyzes of blood and cerebrospinal fluid performed?

 How many PE was done?

 Is hypertension the only reasons of PRES in this case?

 The discussion is too extensive.  

 I see many questions that need to be answered.

Are enough details provided of any physical examination and diagnostic tests, treatment given and outcomes?

No

Is the case presented with sufficient detail to be useful for other practitioners?

Yes

Is sufficient discussion included of the importance of the findings and their relevance to future understanding of disease processes, diagnosis or treatment?

Partly

Is the background of the case’s history and progression described in sufficient detail?

Partly

Reviewer Expertise:

GBS, Status epilepticus, myasthenia gravis crisis

I confirm that I have read this submission and believe that I have an appropriate level of expertise to confirm that it is of an acceptable scientific standard, however I have significant reservations, as outlined above.

# Response

**Published:** 2024-06-26

**Authors:** Koushik Ramachandra

**Affiliations:** General Medicine, Kasturba Medical College, Mangalore, Manipal Academy of Higher Education, Manipal, Mangalore, Karnataka, India

We thank you for valuable suggestions towards improving our case report.

1. Previous Medical History: Our patient did not have medical or neurological history contributing to her current presentation. This has been added to the report.
2. Laboratory investigations: Infection was ruled out with the help of blood counts. Immunological and virological etiologies were also ruled. These have been updated in the report.
3. CSF Tumor markers and Neurology panel:As the NCV findings and clinical profile were favoring a diagnosis of GBS, we did not perform tests for tumor markers.
4. Number of plasma exchanges: Our patient underwent 5 cycles of PE. This has been updated in the report.

# Reviewer report: Reviewer response for version 1

**DOI:** 10.5256/f1000research.161356.r288640

**Published:** 2024-06-24

**Authors:** Amna Al Futaisi

**Affiliations:** 1 Sultan Qaboos University, Al Khoud, Oman

Introduction and Background

 The manuscript presents a rare case of Acute Motor-Sensory Axonal Neuropathy (AMSAN), a severe form of Guillain-Barré Syndrome (GBS), occurring in the immediate postpartum period.

 Novelty and Contribution to Knowledge

 The case report contributes an unusual and severe presentation of GBS in the postpartum period, complicated by PRES. This dual complication is rare and provides valuable insights into the clinical course, management, and outcomes of such cases.

 Clinical Data and Discussion

 The clinical data presented is comprehensive and detailed. The timeline of symptoms, clinical findings, and interventions is well-documented. The inclusion of figures (e.g., Nerve Conduction Studies and MRI findings) adds clarity and supports the diagnostic process.

 However, the discussion could benefit from a deeper analysis of the pathophysiological mechanisms linking GBS, dysautonomia, and PRES. While the manuscript mentions the possible role of autonomic dysfunction and vasogenic edema in PRES, a more detailed exploration of these mechanisms would strengthen the discussion. Additionally, comparing this case with similar reported cases could provide a broader context and highlight the uniqueness of the presented case.

 English Language and Writing Quality

 The manuscript is generally well-written, but there are several areas where the English language could be improved for clarity and readability:

1. Grammar and Sentences: Some sentences are complex and could be simplified. For instance, "Prevention of severe axonal damage in the early stages of the disease stays top priority, it being a major impediment to attaining a favourable long-term outcome" could be revised for clarity.
2. Consistency: Terms such as "post-partum" and "postpartum" should be consistently used throughout the manuscript.
3. Technical terms: Anticonvulsants is outdated and should use antiseizure medications

Adequacy of Clinical Data and Discussion

 The clinical data provided is adequate and detailed, ensuring a clear understanding of the patient's condition, diagnosis, and treatment. The discussion aptly highlights the clinical management and the importance of timely intervention. However, the discussion would benefit from:

1. Including more comparisons with existing literature on similar cases to provide context and underscore the significance of the presented case.
2.  A more thorough exploration of the mechanisms leading to the complications observed in the patient, supported by relevant literature.

Recommendations for Improvement

1. Expand the Discussion: Enhance the discussion section by including more detailed explanations of the pathophysiological mechanisms involved and comparisons with other reported cases.
2. Language Refinement: Revise the manuscript for grammar, syntax, and consistency to improve readability.
3. Conclusion: Strengthen the conclusion to emphasize the key learning points from the case and the implications for clinical practice.

Conclusion

 The manuscript presents a valuable case report with significant clinical insights into the rare and severe presentation of AMSAN variant GBS complicated by PRES in the postpartum period. With improvements in the discussion and language, the manuscript could make a contribution to the medical literature. It is suitable for publication pending major revisions that address the identified areas for enhancement and focusing on the link between PRES and GBS and its pathophysiological explanations. May provide a review on the treatment guideline sin such cases.

  May utilize the following references in the manuscript- (Sorti B. et al., 2020) Ref-1, (Chakraborty T et al., 2020) Ref-2 and (Kobayashi H., 2023) Ref-3.

Are enough details provided of any physical examination and diagnostic tests, treatment given and outcomes?

Yes

Is the case presented with sufficient detail to be useful for other practitioners?

Yes

Is sufficient discussion included of the importance of the findings and their relevance to future understanding of disease processes, diagnosis or treatment?

No

Is the background of the case’s history and progression described in sufficient detail?

Yes

Reviewer Expertise:

Pediatric neurology

I confirm that I have read this submission and believe that I have an appropriate level of expertise to confirm that it is of an acceptable scientific standard, however I have significant reservations, as outlined above.

## References

[1] : Posterior reversible encephalopathy and Guillain-Barré syndrome: which came first, the chicken or the egg? A review of literature. *Neurol Sci* .2020;41(12) : 10.1007/s10072-020-04496-1 3663-3666 10.1007/s10072-020-04496-1 32506357

[2] : Dysautonomia in Guillain-Barré Syndrome: Prevalence, Clinical Spectrum, and Outcomes. *Neurocrit Care* .2020;32(1) : 10.1007/s12028-019-00781-w 113-120 10.1007/s12028-019-00781-w 31297663

[3] : Guillain-Barré Syndrome Complicated by Posterior Reversible Encephalopathy Syndrome and Reversible Cerebral Vasoconstriction Syndrome. *Eur J Case Rep Intern Med* .2023;10(10) : 10.12890/2023_003949 003949 10.12890/2023_003949 37789979 PMC10545146

# Response

**Published:** 2024-06-26

**Authors:** Koushik Ramachandra

**Affiliations:** General Medicine, Kasturba Medical College, Mangalore, Manipal Academy of Higher Education, Manipal, Mangalore, Karnataka, India

We thank you for valuable suggestions towards improving our case report.

1. Language and Grammar: As suggested by you, the language and grammar of the entire report has been reworked. Older, outdated terminologies have been replaced.
2. Pathophysiologic understanding: We thank you profusely for sharing 3 articles for us to use. We have tried to incorporate their findings, also comparing them with our case.
3. Conclusions: This section has been reworded to shed light on the importance of dysautonomia in GBS.
